# Case of Cellulo-vascular and Fibrous Polypus Occurring Simultaneously; Removal of Both by the Ligature

**Published:** 1846-11

**Authors:** John F. Meigs

**Affiliations:** Lecturer on Midwifery, &c., in the Philadelphia Medical Association


### MEDICAL EXAMINER

AND

### RECORD OF MEDICAL SCIENCE.

NEW SERIES.—No. XXIII.—NOVEMBER, 1846.

### ORIGINAL COMMUNICATIONS.

*Case of cellulo-vascular and fibrous Polypus occurring simultaneously; removal of both by the ligature.* By JOHN F. MEIGS, M. D., Lecturer on Midwifery, &c., in the Philadelphia Medical Association.

Mrs. J. F. the subject of the following case, is an Irish woman about forty years of age. She is the mother of several children. Her last pregnancy terminated in a miscarriage on the 24th of April, 1843. About the end of the year 1843, or the beginning of 1844, (she cannot recollect which), she began to be subject to violent and frequently repeated attacks of uterine hemorrhage, which exhausted her very much.

I was first called to see her on the 20th of January, 1845. She had then been flooding very badly for several days, and was extremely weak; she had fainted several times, and seemed almost exsanguious; she had been under the charge of several physicians, by whom she had been treated with various astringent remedies.

I made a vaginal examination at once, and found two polypi growing from the cervix uteri; one was about two inches long, pyriform in shape, with a base about an inch and a half wide, rapidly narrowing to a small slender pedicle, which was attached to the cavity of the cervix uteri; this one was soft and yielding to the touch. The other tumour was much larger, and grew from the posterior lip of the os uteri; it seemed indeed to be entirely a production of that lip. The shape was pyriform like that of the other, but the pedicle was much larger in proportion, and the whole tumour seemed to be about twice as large. Its consistence was entirely different; it was hard, resisting and somewhat elastic to the touch; it had in fact all the characters of a hard, fibrous polypus. It was about as large as a full sized butter pear, while the smaller one was rather less than a well-grown catherine pear.

On the 22d of January, 1845, aided by Dr. Mason of this city, I passed a ligature, by means of Gooch's double canula, around the pedicle of the smaller polypus. It was somewhat difficult to adjust the loop, in consequence of the presence of the second tumour in the vagina, but after a little patient manipulation, I succeeded in fixing it high up about the pedicle. On tightening the ligature the polypus fell off; it proved to be a cellulo-vascular polypus; no hemorrhage followed its separation, and the patient did not complain of any pain.

It was thought best not to operate upon the second tumour for some time. The patient was for several weeks in a very critical situation; she had symptoms of peritoneal inflammation, which however yielded to very simple treatment. After this she was attacked with fever of a low type, which resulted at last in an extensive purulent collection over the posterior surface of the sacrum. This was opened, and she gradually regained a pretty good state of health; she regained strength enough to go about the house and attend to the cares of her family.

She had some slight attacks of hemorrhage in the latter part of 1845, and in the early months of the present year.

Sometime in May of the present year, she was seized with a most violent flooding, which for some time exposed her life to great danger. After this I requested Dr. Hodge to visit her with me, in order to determine upon the propriety of removing the large polypus, which had increased very much in size. At this time it filled the whole cavity of the vagina, and during the efforts of defecation and urination, frequently passed partly through the vulva. Having agreed upon the operation, I proceeded to perform it, with the aid of my friend, Dr. William Keating, who also assisted me in the after treatment of the case.

Thursday, June 11th, 1846,—At 5 o'clock P. M., I applied, by means of Gooch's double canula, a large strong ligature of twisted silk around the pedicle of the tumour, at the distance of about a quarter of an inch from the cervix uteri. As the tumour grew from the whole of the posterior lip of the os uteri, I was careful not to include any portion of the substance of the uterus in the grasp of the ligature. The ligature was drawn as tightly at the time as it could be drawn, and then carefully secured. It gave but little pain. The patient was desired to lie chiefly on her side, and to be as quiet as possible. As she was very much exhausted from her frequent attacks of hemorrhage, she was allowed to take pretty freely of light, nutritious diet, and from time to time she took small quantities of milk punch. She was to have an opiate in the evening.

As no bad symptoms occurred, the ligature was tightened every day. In three or four days, the discharge from the vagina became very offensive, and on tightening the ligature, it was evident that it cut through a portion of softened tissue.

No bad symptoms arose for several days, except the occurrence of violent abdominal pain, with some tympany and soreness to the touch, on one occasion, after the ligature had been drawn very tight. These symptoms, however, disappeared after loosening the instrument a little, and left no bad consequences.

On Saturday, the 20th, there was inability to discharge the urine. The difficulty seemed to depend upon the pressure of the instrument and the tumour upon the urethra. The tumour had increased very much in size, so as to fill and distend the whole of the vagina. The external parts were very much swelled and irritated, and they as well as the vagina had become painful to the touch. The catheter was introduced with some little difficulty, room being obtained by pushing the canula backwards towards the perineum. The catheter was used on the 20th, 21st and 22d.

On Sunday, June 21st, ten days after the application of the ligature, the tumour was forced through the vulva, during some effort the patient was making. It was about as large as a large ostrich egg. Its external surface was rough and uneven, and of a deep red colour. It was hard and fibrous to the feel, and looked very much indeed like a piece of coarse flesh. The pedicle, which was closely embraced by the ligature, was not more than two or three lines in thickness. As it gave the patient no greater inconvenience in its present situation than it did before it was protruded, it was allowed to remain.

At a little after two o'clock, on Monday the 22d, the tumour fell off from its attachments, the separation taking place about half an inch above the point embraced by the ligature. The ligature with the canula remained fastened upon the tumour. The ligature had remained, therefore, very nearly eleven days, before the vitality of the polypus was sufficiently destroyed to cause it to drop off.

The polypus upon examination, proved to be very large. It was between four and five inches long, by three or four broad. It would about fill a large ostrich egg. It was fleshy in consistence, of a sanguine red colour, rough upon the surface, of homogeneous texture, without any cavity, and presented in fact all the characteristics of a hard, fibrous polypus.

The patient was weak and fatigued from the constraint and irritation she had undergone during the operation of the ligature, but had no other bad symptoms.

July 2d, 1846.—She has had no trouble since the separation of the polypus, so far as regards the pelvic organs, with the exception of slight ardor urinæ. Her command over the bladder is perfect. The bowels act well, and she has had no symptoms of inflammation of the uterus, ovaries or peritoneum.

She has had for a week past some teasing cough, with a little hectical irritation. She complains of aching, erratic pains affecting the left side of the thorax. There is some dulness of percussion over the lower third of the left lung, behind, with feeble respiratory murmur, and slight crepitus after coughing. The expectoration, which is small in quantity, is of thick colourless mucus. She is to take a few grains of iodide of potassium in compound syrup of sarsaparilla, three times a day, and to have a Burgundy pitch plaster applied to the side.

On making a vaginal examination I found the shape of the os uteri well preserved. The direction of the opening is transverse, and its width about an inch. The margin of the vaginal portion of the cervix is very well marked; even the posterior lip itself retains its proper form, with the exception of a slight irregularity. The whole of the cervix uteri was swelled, and felt much harder than in the ordinary condition, though there was nothing like scirrhous hardness or irregularity that I could detect.

As she was able now to move about her room a little, and seemed to be contending only with weakness and the pleuritic affection, we recommended her to go to the country for a short time to recruit.

Early in August, after she had been two weeks in the country, I saw her again. She had recovered, in a great degree, good health. She had become quite strong; went freely about the house, had a good appetite, had lost all her pulmonary symptoms, was fast losing the anemic look which had so long set its mark upon her, and was in fact restored apparently to good health. She had not had, at any time since the operation, any symptoms of disease of the reproductive organs.

